# Effect of Coagulant and Treatment Conditions on the Gelation and Textural Properties of Acidic Whey Tofu

**DOI:** 10.3390/foods12050918

**Published:** 2023-02-21

**Authors:** Ziyu Guan, Jie Zhang, Shitong Zhang, Yun He, Yadi Li, Joe M. Regenstein, Yuan Xie, Peng Zhou

**Affiliations:** 1 State Key Laboratory of Food Science and Technology, Jiangnan University, Wuxi 214122, China; 2 School of Food Science and Technology, Jiangnan University, Wuxi 214122, China; 3 Department of Food Science, Cornell University, Ithaca, NY 14853-7201, USA

**Keywords:** acidic whey coagulant, *Lactiplantibacillus paracasei*, *Lactiplantibacillus plantarum*, rheological properties, texture, tofu gelation

## Abstract

This study aimed to investigate the properties of acidic whey tofu gelatin generated from two acidic whey coagulants by pure fermentation of *Lactiplantibacillus paracasei* and *L. plantarum*, as well as the characteristics of acidic whey tofu. The optimal holding temperature and the amount of coagulants added were determined based on the pH, water-holding capacity, texture, microstructure, and rheological properties of tofu gelation. Then, the differences in quality between tofu produced by pure bacterial fermentation and by natural fermentation were investigated under optimal tofu gelatin preparation conditions. The tofu gelatin presented the best texture at 37 °C with a 10% addition of coagulants fermented by both *L. paracasei* and *L. plantarum*. Under these conditions, the coagulant produced by the fermentation of *L. plantarum* resulted in a shorter formation time and stronger tofu gelatin compared with that produced from *L. paracasei*. Tofu produced by the fermentation of *L. paracasei* had higher pH, less hardness, and a rougher network structure, whereas tofu produced by the fermentation of *L. plantarum* was closer to tofu produced by natural fermentation in terms of pH, texture, rheology, and microstructure.

## 1. Introduction

Tofu is a highly hydrated soybean protein gelatin, and the essence of its production process is the extraction and gelation of soybean protein [1,2]. After screening and cleaning, impurity-free soybeans are obtained, which are fully soaked in water and then ground to obtain raw soybean milk. After soybean milk is boiled, the coagulant of appropriate concentration is added. The product is pressed into shape, and the excess liquid is discharged, which is soybean whey [3]. In traditional Chinese tofu production, acidic whey, which is produced by the natural fermentation of soybean whey, was often used as the coagulant to make acidic whey tofu [4,5]. When coagulant is added to cooked soybean milk, the lactic acid bacteria in it use the carbohydrates in soybean milk to produce large amounts of lactic acid, causing the pH of the solution to drop and move toward the isoelectric point of the soybean protein [6,7]. In this acidic solution, the decreasing protein surface electrical charge further reduces the electrostatic repulsion on the protein surface, destroying the colloidal stability and forming soybean protein gelatin by protein cross-linking under the action of hydrogen bonding and hydrophobicity [8]. However, the production of acidic whey tofu is mostly home-based. The judgment of the fermentation ending point of coagulant is mostly based on manual experience and lacks scientific guidance. In addition, coagulant is generally obtained by the natural fermentation of soybean whey placed outdoors, which may contain harmful microorganisms. Moreover, in actual production, the coagulant is easily affected by the environment and season, and the quality of the coagulant varies greatly from batch to batch [4,9]. Therefore, the variation in the quality of coagulant leads to the unstable quality of acidic whey tofu, seriously restricting the development of tofu.

As people have become more health conscious in recent years, tofu has become a new trend as a new lactic acid bacteria carrier. Chen et al. [10] used *Weisseria greekii* D1501 in combination with transglutaminase to produce a new type of lactic acid bacteria tofu that was creamy, smooth, soft, and elastic, which was effective in inhibiting contamination by other bacteria and extending the shelf life of tofu. Riciputi et al. [11] and Serrazanetti et al. [12] used *L. casei* and *L. acidophilus* to ferment soybean milk and salt brine as tofu coagulants and found that *L. acidophilus* could produce enough substances such as acetic acid, limonene, and benzyl alcohol to inhibit the growth of spoilage bacteria while promoting the synthesis of bioactive substances in tofu. Wang et al. [13] used *L. plantarum* fermented plum juice as an acidic coagulant for tofu and produced tofu with plum juice that was close to the quality of lactic tofu and could be stored for 1 week at 4 °C.

This study aimed to determine the properties of tofu gelatin and tofu produced from two acidic whey coagulants by pure fermentation of *L. paracasei* and *L. plantarum*. Our study provided theoretical guidance for producing stable and good-quality acidic whey tofu, which was conducive to the industrial production of local specialty curd.

## 2. Materials and Methods

### 2.1. Materials

Soybeans were purchased from Anhui Huaibei Jinyuan Soybeans Wholesale Co., Ltd. (Huaibei, Anhui, China). Acidic whey was collected from a local tofu manufacturing market and stored in −20 °C for future use. *L. plantarum Lb-p1* and *L. paracasei* were obtained from acidic whey collected from Chuxiong, Yunnan, China.

The processing procedure for the manufacture of acidic whey tofu is shown in Figure S1. High-quality soybeans with whole kernels were selected and soaked at room temperature for 10–12 h at a soybean:water ratio of 1:3.5. The soybeans were then drained and pulverized in a hot-wall cooker for 1 min at a soybean:water ratio of 1:8 (water added = dry soybean mass × 9—soaked soybean mass). The soybean milk was filtered through 120-mesh gauze. The soybean milk was heated in a boiling water bath and cooled in an ice water bath after the heating was completed.

### 2.2. Preparation of Acidic Whey Tofu Gelatin and Acidic Whey Tofu

The acidic whey coagulant was obtained by inoculating *Lb-p1* (6 log CFU mL^−1^) or *L. paracasei* in sterile 0.85% NaCl into sterile soybean milk with a volume percentage of 3% and then cultivating at 37 °C for 12 h. For optimizing the temperature, this coagulant was added to soybean milk at the ratio of 1:10 (*v*/*v*) and incubated at 27, 37, 47, 57, and 67 °C for 6 h using the water bath. For optimizing the ratio, the coagulant was mixed with soybean milk with the percentage of 4%, 7%, 10%, 13%, and 16% and incubated at 37 °C for 6 h using the water bath. The obtained acidic whey tofu gelatin was cooled down using ice water to 4 °C for analysis.

Soybean milk (500 mL) was taken, and 50 mL of pure-bacterial-fermented coagulant and naturally fermented coagulant were added separately and kept warm for 6 h. The mixture was poured into a tofu mold (8 × 8 × 6 cm^3^) and pressed with a force of 25 g/cm^2^ for 90 min to obtain different acidic whey tofu, which was stored in a refrigerator at 4 °C for backup.

### 2.3. Determination of the pH Value of Acidic Whey Tofu Gelatin and Acidic Whey Tofu

Acidic whey tofu gelatin (10 g) was weighed and pounded, and its pH was measured directly with a pH meter (Mettler Toledo, Columbus, OH, USA).

After adding deionized water (20 mL) to the acidic tofu (5 g), it was homogenized with a disperser for 1 min and then stirred at room temperature for 30 min. The pH of the sample was measured directly using the pH meter.

### 2.4. Determination of the Water-Holding Capacity of Acidic Whey Tofu Gelatin and Acidic Whey Tofu

After the soybean milk was mixed with the acidic whey, it was placed in a 50 mL centrifuge tube, kept in a water bath for 6 h, and left overnight at 4 °C. After reaching room temperature, the sample was centrifuged at 20 °C for 10 min at 4000× *g*. The water left on the bottom of the tube was poured out, and the tube was placed upside down on filter paper to absorb the water flow. The water-holding capacity of the acidic whey tofu gelatin was defined as the ratio of the mass of the sample after centrifugation to the mass of the sample before centrifugation.

For acidic whey tofu, its water-holding capacity was determined using the same method.

### 2.5. Determination of Texture Profile Analysis of Acidic Whey Tofu Gelatin and Acidic Whey Tofu

Acidic whey tofu gelatin stored overnight at 4 °C was removed and equilibrated at room temperature for 1 h. The hardness of gelation was determined using TA-XT Plus (Stable Micro Systems, Ltd., Godalming, UK). The measurement conditions were as follows: a P/0.5 R probe was selected, the pre-test speed, mid-test speed, and post-test speed were 0.8 mm/s, the trigger force was 1 g, and the measurement distance was 6 mm. The probe was centered on the sample, and the hardness of gelatin was expressed as the maximum induced force (g) obtained during the determination.

Acidic whey tofu stored overnight at 4 °C was cut into blocks of 2 × 2 × 2 cm^3^ and equilibrated at room temperature for 1 h. Texture profile analysis (TPA) was determined using TA-XT Plus. The measurement conditions were as follows: P50 probe was selected, and the premeasurement velocity, mid-measurement velocity, and post-measurement velocity were 1.5 mm/s, 1 mm/s, and 1 mm/s, respectively; the trigger force was 2 g; and the measurement distance was 5 mm. The probe was centered on the sample, and the tofu gelation hardness was expressed as the maximum induction force (g) obtained during the measurement [14].

### 2.6. Determination of the Rheological Properties of Acidic Whey Tofu Gelatin and Acidic Whey Tofu

Rheological properties were measured with a HAAKE MARSⅢ rheometer (Thermo Fisher Scientific Inc., Waltham, MA, USA). For acidic whey tofu gelatin, temperature scanning was applied using a 50-mm parallel-plate probe with a gap setting of 1 mm. A strain of 1% (within the linear range) and a frequency of 1 Hz were selected. Soybean milk (10 mL) was mixed with an appropriate coagulant, and the sample was quickly added to the sample stage. The rheometer probe was lowered, and the excess sample around the probe was aspirated. The sample was then sealed with silicone oil and solvent cap to avoid water evaporation during the measurement. The experimental procedures for temperature scanning were as follows: the warming program scan was 25–37 °C at the rate of 2 °C/min, the constant-temperature program scan was 37 °C for 360 min, and the cooling program scan was 37–25 °C at the rate of 2 °C/min. The trends of the storage modulus (G’) and lost modulus (G’’) with time during the temperature scan were recorded. After the temperature scan, acidic whey tofu gelatin was subjected to a frequency scan at 25 °C with an angular frequency scan range of 1 rad/s to 100 rad/s and a strain of 1%. The trends of G’ and G’’ with the angular frequency scan range during the temperature scan were recorded [15].

Acidic whey tofu was determined using both strain creep response and creep-recovery response. For strain creep response, the sample was cut into thin slices of about 2 mm, and the strain was gradually increased from 1% to 200% at 25 °C using a 25 mm parallel-plate probe with a gap setting of 2 mm and a frequency of 1 Hz. For creep-recovery response, Shear stress (8 Pa) was applied to the sample at 25 °C, and the change in strain with time was recorded for 5 min. Then, the stress was removed, and the change in strain with time was continuously recorded for 5 min [6,16,17].

### 2.7. Analysis of Microstructure of Acidic Whey Tofu Gelatin and Acidic Whey Tofu

Acidic whey tofu gelatin and tofu was cut into thin slices of approximately 1.5 mm thickness and placed in 2.5% glutaraldehyde solution (0.1 M, pH 7.4, in phosphate buffer configuration) for 36 h. The samples were rinsed 3 times with phosphate buffer for 10 min each time. The samples were further dehydrated with an ethanol gradient (30%, 50%, 60%, 70%, 80%, and 90%) for 20 min each time and then dehydrated twice with anhydrous ethanol for 30 min each time. The dehydrated samples were replaced twice with tert-butanol for 30 min each. Finally, the samples were frozen with liquid nitrogen (−196 °C) and then vacuum freeze-dried. The dried samples were broken off and subjected to ion-sputtering gold spraying. The cross-sectional structure was observed using scanning electron microscopy (SEM) [18,19,20].

### 2.8. Determination of the Organic Acids in Acidic Whey Tofu

After adding 20 mL of deionized water into the acidic whey tofu (5 g), the mixture was homogenized with a disperser for 1 min and stirred at room temperature for 30 min. The sample was centrifuged at 10,000× *g* for 10 min, following which the supernatant was collected and filtered through a 0.22 m membrane. The collected liquid was analyzed using high-performance liquid chromatography (Agilent 1200, Agilent Technologies, Inc., Santa Clara, CA, USA) with a Diamonsil C18 column (4.6 mm × 250 mm, 5 μm, Dikma Technologies Inc., Lake Forest, CA, USA). The mobile phase was methanol: water: phosphoric acid (5:95:0.05, volume ratio), and the flow rate was 0.6 mL/min at 30 °C.

### 2.9. Statistical Analysis

All data were analyzed using the Statistical Package for the Social Sciences, SPSS 20 (IBM Corp., Chicago, IL, USA). The results are given as mean ± standard deviation (SD), significant differences in mean values were determined using Duncan’s multiple range tests, *p* values of <0.05 were considered to be significant, and *p* < 0.01 were very significant.

## 3. Results and Discussion

### 3.1. Characterization of pH and Organic Acids during the Fermentation of Soybean Whey by Lactic Acid Bacteria

**Figure 1:**
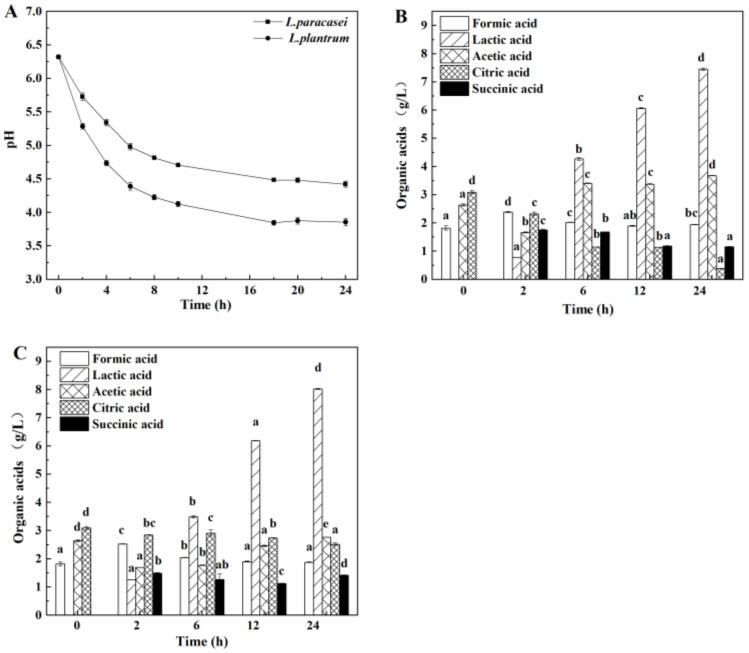
Changes of pH during the fermentation of acidic whey using *L. paracasei* and *L. plantarum* (**A**). Changes of organic acids in acidic whey during the fermentation using *L. paracasei* (**B**) and *L. plantarum* (**C**). Note: Different letters of the same column filling mean significant differences (*p* < 0.05).

The changes in the pH value during the fermentation of soybean whey by *L. paracasei* and *L. plantarum* were compared to obtain lactic acid bacteria that could use soybean whey with high acid production capacity. The results are shown in Figure 1A. The pH value of soybean whey decreased gradually as the fermentation process proceeded. The pH value decreased rapidly during the first 8 h of fermentation and then changed slowly. Compared with *L. paracasei*, the pH value of *L. plantarum* changed faster during the fermentation of soybean whey. The pH value was as low as 3.85 at the end of fermentation, indicating that *L. plantarum* produced acid faster and had a stronger acid production ability than *L. paracasei*.

The changes in five organic acids, namely formic acid, lactic acid, acetic acid, citric acid, and succinic acid, during the fermentation of soybean whey by *L. paracasei* and *L. plantarum* are shown in Figure 1B,C, respectively. After soybean whey was fermented by *L. paracasei* and *L. plantarum* for 24 h, the total amount of the 5 organic acids increased substantially, which was consistent with the gradual decrease in pH value in soybean whey during the fermentation process. As shown in Figure 1B, the total amount of the 5 organic acids increased from 7.55 g/L to 14.61 g/L after 24 h of fermentation of soybean whey by *L. paracasei*, among which the lactic acid content increased from 0 g/L at the beginning to 7.45 g/L, accounting for 50.99% of the total acid content. However, the content of citric acid decreased, probably due to the consumption of bacteria, especially when *L. paracasei* was used. After the 24 h fermentation of soybean whey by *L. plantarum*, the total amount of the 5 organic acids increased from 7.55 g/L to 16.59 g/L, among which the lactic acid content increased from 0 g/L at the beginning to 8.02 g/L, accounting for 48.34% of the total acid content. In general, lactic acid and acetic acid were mainly produced during the fermentation of soybean whey by *L. paracasei* and *L. plantarum*. After 24 h of fermentation, the total amount of organic acids in soybean whey fermented by *L. plantarum* was higher than that of *L. paracasei*.

### 3.2. Acidic Whey Tofu Gelatin

#### 3.2.1. Effect of Holding Temperature on the Gelation Condition, pH, Hardness, Water-Holding Capacity, and Microstructure of Gelatin

The acidic whey prepared by the fermentation of *L. paracasei* and *L. plantarum* was used as a coagulant for tofu manufacture and held at 27 °C, 37 °C, 47 °C, 57 °C, and 67 °C for 6 h. The solidification of soybean milk is shown in Figure S2. Using acidic whey formed by *L. paracasei* fermentation, the acidic whey tofu gelation was formed only at 37 °C and 47 °C, while soybean milk remained in the liquid state at 27 °C, 57 °C, and 67°C. In contrast, using the acidic whey formed by *L. plantarum* fermentation, the tofu gelation was formed at all five holding temperatures.

The properties of tofu gelatin prepared from coagulant fermented from *L. paracasei* or *L. plantarum* were determined. The results are shown in Table 1. Using coagulant fermented by *L. paracasei* and *L. plantarum*, the pH of tofu gelatin showed a tendency to decrease and then increase with the increasing holding temperature when the amount of coagulant added was 10%. At the same temperature (except 67 °C), the pH of tofu gelatin made with the coagulant fermented with *L. plantarum* was lower than that of tofu gelatin made with the coagulant fermented with *L. paracasei*. When the holding temperature was 37 °C, the pH value of tofu gelatin made with coagulants fermented with *L. paracasei* and *L. plantarum* reached the lowest value of 5.64 and 4.50, respectively. As the holding temperature increased, the water-holding capacity of tofu gelatin was the lowest at the holding temperature of 37 °C with the coagulant fermented with *L. plantarum*. At different holding temperatures, the hardness of tofu gelatin showed a trend of increasing and then decreasing with the coagulant fermented with *L. plantarum*. The hardness of tofu gelatin reached its maximum at the holding temperature of 37 °C, and the hardness of tofu gelatin made from *L. plantarum* was 2.5 times higher than that made from *L. paracasei*.

**Figure 2:**
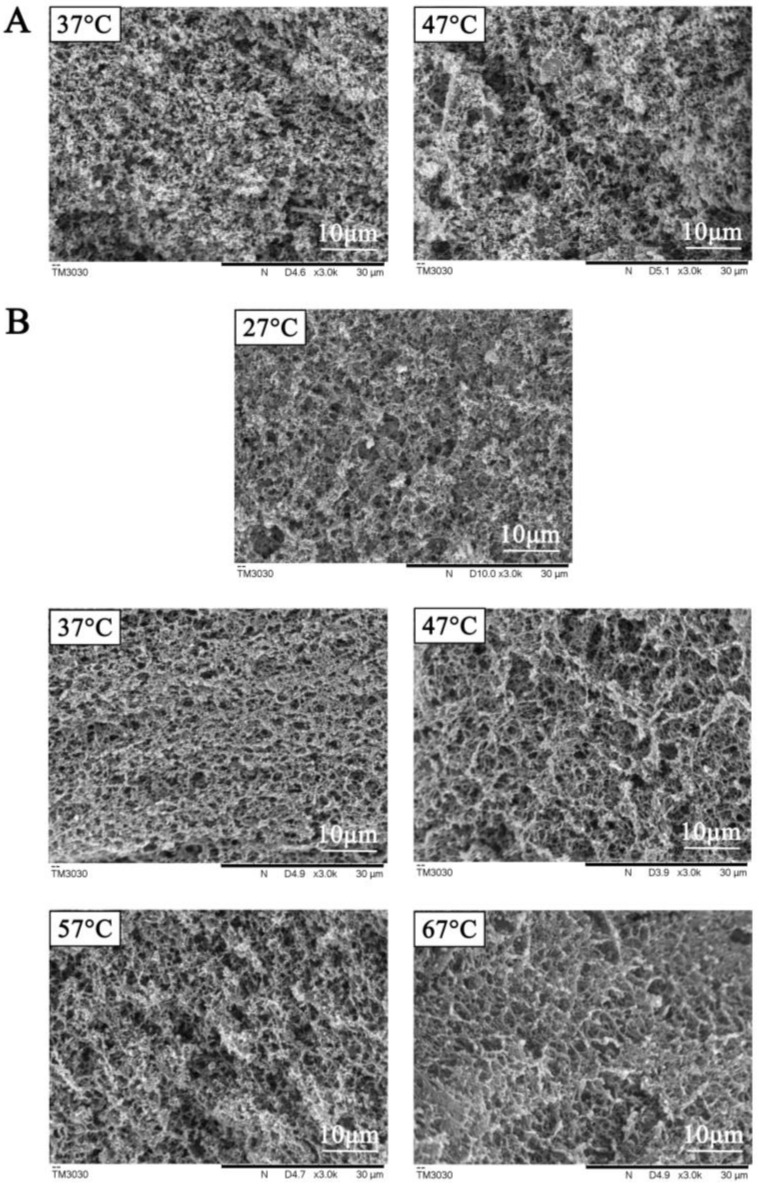
SEM images of tofu gelatin induced by coagulant fermented using *L. paracasei* (**A**) and *L. plantarum* (**B**) at different temperatures.

The microstructure of tofu gelatin using coagulant fermented with both bacteria at different holding temperatures was observed by SEM (Figure 2). The pores of tofu gelatin formed by *L. paracasei* at 37 °C were uniform and dense. The tofu gelatin formed at a holding temperature of 47 °C had larger pores and a heterogeneous structure. However, tofu gelatin made from *L. plantarum* had a more uniform and orderly network structure than *L. paracasei*. The pores of tofu gelatin were smaller and more uniformly dense at the holding temperature of 37 °C than other temperatures.

The tofu gelatin with a low pH value, high hardness, and a uniform and dense structure was obtained at the holding temperature of 37 °C using both *L. paracasei*- and *L. plantarum*-fermented coagulants. It might be because both *L. paracasei* and *L. plantarum* were able to grow and multiply better at 37 °C and facilitate acid production, thus promoting the slow binding of soy protein, achieving the best coagulation rate, and having the ability to encapsulate water, fat, and small-molecule proteins into the protein network structure to form a more orderly network structure. When the holding temperature was higher than 37 °C, the growth and reproduction of lactic acid bacteria were affected. Furthermore, the thermal movement between protein molecules was too fast, and the chances of mutual collision and binding were higher. The protein solidification rate is accelerated, resulting in a tofu gelatin network structure with larger holes and reduced hardness. When the holding temperature was lower than 37 °C, the growth of lactic acid bacteria and the protein movement were slower. Therefore, the gelation process might occur throughout the holding process, but the protein coagulation was slower, resulting in a weaker gelatin structure.

#### 3.2.2. Effect of Coagulant on the Gelation Condition, pH, Hardness, Water-Holding Capacity, and Microstructure of Gelatin

The properties of tofu gelatin produced by different coagulant additions are shown in Table 2. The solidification of soybean milk is shown in Figure S3. The pH of tofu gelatin decreased with the increase in the addition of a coagulant after 6 h of incubation. The pH of tofu gelatin started to approach 4.5 when the addition of the coagulant fermented with *L. plantarum* was higher than 10%. Using the coagulant fermented with *L. paracasei*, the pH of tofu gelatin was still above 5.5 when the coagulant was added at 16%. The water-holding capacity of tofu gelatin showed an overall decreasing trend with the addition of the coagulant, using coagulants fermented with both *L. paracasei* and *L. plantarum*. The hardness of tofu gelatin made from *L. plantarum* reached a maximum with a value of 57.22 g after a 10% addition of coagulant.

**Figure 3:**
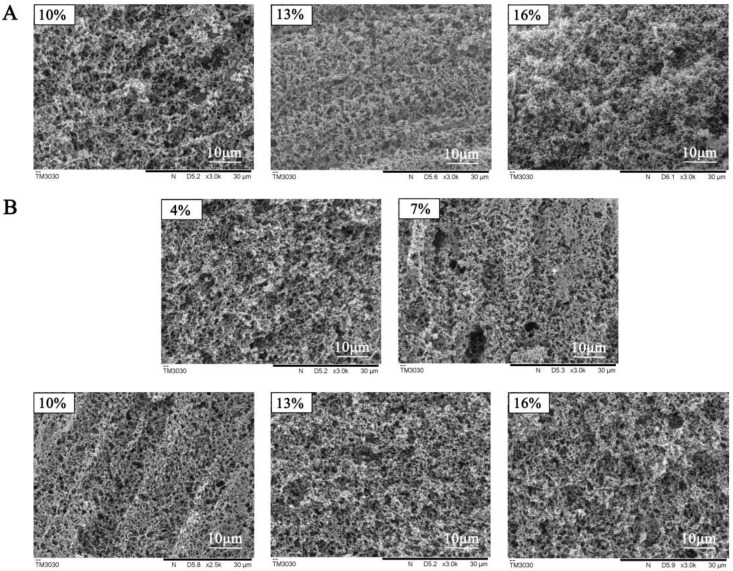
SEM images of tofu gelatin induced by different percentage of *L. paracasei* (**A**) and *L. plantarum* (**B**) fermented coagulant.

The microstructure of tofu gelatin with different additions of coagulant fermented with *L. paracasei* and *L. plantarum* was observed by SEM (Figure 3). After adding 10% coagulant fermented with *L. paracasei*, the reticulation structure of tofu gelatin was clearly observed with small, dense, and evenly distributed pores. With the increase in the addition of coagulant, the structure was denser. The structure of tofu gelatin was uneven, and the pores formed were large when the concentration of coagulant fermented with *L. plantarum* was 4% and 7%. When the concentration of this coagulant was 10%, the pores of the tofu gelatin were small and evenly distributed. When the addition of coagulant was further increased, the pores of the tofu gelation were still relatively uniform and dense.

The concentration of coagulant is an important factor affecting the quality of tofu gelatin. When the coagulant concentration is extremely low, the pH of soybean milk is much higher than the soybean protein isoelectric point, with the electrostatic repulsion in soybean protein dominating. Therefore, most proteins cannot cross-link to form a gelation network. When the coagulant concentration increases, the electrostatic repulsion between protein molecules weakens, and the gravitational force increases. Although the proteins can combine to form a gelatin network, the structure of the network is coarse and uneven, and the strength of gelatin is low, which makes it difficult to bind the small molecules. When the coagulant concentration continues to increase, the pH was lower than the protein isoelectric point [21,22]. The gravitational force and the repulsive force between protein molecules reach an equilibrium, forming a uniform and orderly network structure. Other substances, such as small molecules of protein and water, are evenly distributed in the network structure, and the strength of gelation is higher. When the coagulant concentration increases further, the balance between the gravitational and repulsive forces among protein molecules is broken, and H^+^ makes the proteins positively charged, leading to electrostatic repulsion again. This results in the contraction of the gelatin network. The ability of the network to bind proteins and water, as well as the gelatin strength, decreases. When the tofu gelatin network is uniform and dense, it can better bind water, lipids, and other substances in the network structure. Additionally, the tofu gelatin formed is hard, and the yield of tofu is high [23,24].

#### 3.2.3. Study of the Rheological Properties of Acidic Whey Tofu Gelatin

**Figure 4:**
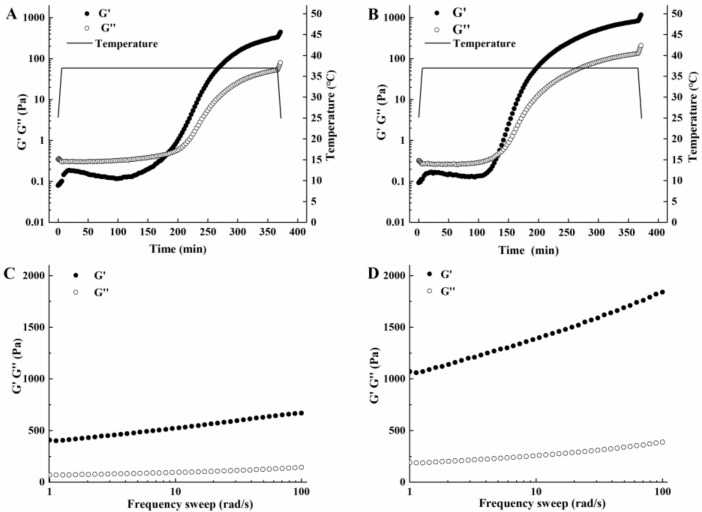
Time sweep G’ and G’’ of tofu gelatin induced by coagulants fermented by *L. paracasei* (**A**) and *L. plantarum* (**B**). Frequency sweep G’ and G’’ of tofu gelatin induced by coagulants fermented by *L. paracasei* (**C**) and *L. plantarum* (**D**).

The acidic whey fermented by *L. paracasei* and *L. plantarum* were used as the coagulants to understand the differences in the rheological properties of tofu gelatin. The variations in storage modulus (G’) and loss modulus (G’’) with time were measured for tofu gelatin at 37 °C with a 10% addition of coagulant (Figure 4). Initially, G’ was smaller than G’’. As time increased, G’ increased rapidly, intersected with G’’, and continued to rise; the intersection point was called the gelation point [25]. Using coagulants fermented by *L. paracasei* and *L. plantarum*, G’ was equal to G’’ in 182.4 min and 132.9 min, respectively, and soybean milk started to gelatinize. Compared to *L. paracasei*, the coagulant fermented by *L. plantarum* required a shorter gelation time. The transformation of tofu gelatin from a liquid dispersion into a solid gelatin structure was completed during the holding process. During the cooling process, G’ and G’’ continued to rise, indicating that the structure of gelatin was gradually enhanced during the cooling process, probably because the cooling process facilitated the formation of hydrogen and ionic bonds, thus increasing the binding interaction between the proteins [18]. It was also observed that the storage modulus G’ was greater with coagulant fermented by *L. plantarum*, indicating greater gelatin strength [26,27], which was consistent with the data of gelatin hardness (Table 2).

Frequency scans were performed on tofu gelatin samples to investigate further the viscoelasticity and force of coagulant-induced soy protein gelatin. As shown in Table S1, the magnitude of n reflected the nature of the forces in gelatin. When n was close to 0, it indicated that gelatin was chemical in nature and consisted of strong covalent bonds. When n was greater than 0, it indicated that the gelation was physical in nature, and the main forces were weak noncovalent bonds [28]. The model fit results (shown in Table S1) with R^2^ greater than 0.99 indicated a good fit. Using coagulants fermented by *L. paracasei* and *L. plantarum*, the n value of tofu gelatin was 0.1156 and 0.1220, respectively, which was greater than 0, indicating that the tofu gelatin had weak physical gelatin. In addition, a correlation existed between the n value and gelatin strength. The larger the n value, the stronger its gelatin strength, which was consistent with the value of gelation endpoint G’.

In summary, using coagulants fermented by *L. paracasei* and *L. plantarum* and comparing the pH value, water-holding capacity, hardness, and microstructure of tofu gelatin, a better quality of tofu gelatin was obtained when the amount of coagulant added was 10% and the holding temperature was 37 °C.

### 3.3. Evaluation of the Quality of Acidic Whey Tofu

#### 3.3.1. Effect of pH, Water-Holding Capacity, Texture, and Structure of Acidic Whey Tofu Fermented Using Different Strains of Bacteria

The acidic whey tofu production process requires pressure drainage to drain water from the tofu gelatin and reconstruct the texture. Pressurized water drainage can facilitate the binding of dispersed protein gelatin inside tofu, resulting in a more compact tofu structure. Naturally fermented tofu was added as a control to evaluate the difference in the quality of acidic whey tofu produced using coagulants fermented by *L. paracasei* and *L. plantarum* and naturally fermented acidic whey.

The pH and water-holding capacity of tofu produced from the *L. paracasei* group, the *L. plantarum* group, and the naturally fermented acidic whey group are shown in Table 3. The pH value of tofu in the *L. plantarum* group was 4.64, and that in the naturally fermented group was 4.23, which were close to each other, while the pH value of tofu in the *L. paracasei* group was much higher at 5.62. The water-holding capacity was higher in the *L. plantarum* group, followed by the *L. paracasei* group, and the lowest in the naturally fermented group, indicating that the tofu of the *L. plantarum* group had a higher ability to bind free water.

Table 3 provides the values of hardness, elasticity, cohesiveness, and chewiness parameters corresponding to the whole mass composition. As shown in Table 3, the elasticity of tofu in the *L. paracasei* group, the *L. plantarum* group, and the naturally fermented group was similar, which was 0.93, 0.92, and 0.93, respectively. The cohesiveness of tofu in the *L. paracasei* group was greater than that of tofu in the *L. plantarum* group and tofu in the naturally fermented acidic whey group. The hardness of tofu in the *L. plantarum* group and the naturally fermented group was similar, which was higher than the hardness of tofu in the *L. paracasei* group. Chewiness positively correlated with hardness, and the trend of chewiness variation and hardness variation in the three types of tofu was consistent. The hardness, elasticity, chewiness, and cohesiveness of tofu in the *L. plantarum* group were comparable to those in the naturally fermented group, indicating that the two types of tofu had similar structural properties.

**Figure 5:**
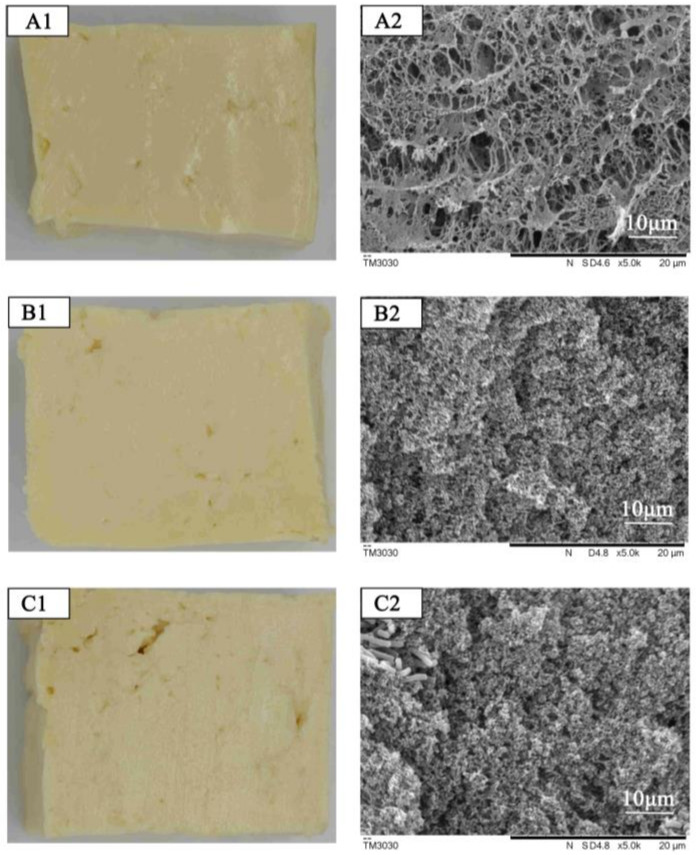
Pictures of tofu manufactured from coagulants fermented by *L. paracasei* (**A1**), *L. plantarum* (**B1**), and natural bacteria (**C1**). SEM images of tofu manufactured from coagulants fermented by *L. paracasei* (**A2**), *L. plantarum* (**B2**), and natural bacteria (**C2**).

Figure 5 showed the appearance and microstructure of tofu manufactured from coagulants fermented by *L. paracasei*, *L. plantarum*, and natural bacteria. From the appearance, the tofu gelatin in the *L. paracasei* group adhered as a whole after pressing, with water overflowing from the surface of the tofu and having a smooth cut surface. The tofu in the *L. plantarum* group and the tofu in the naturally fermented group adhered during the pressing process, but gaps were created during the pressing process. The internal microstructure of the three groups of tofu was observed using SEM. The tofu in the *L. paracasei* group was relatively rough and porous, having holes of different sizes and an uneven structure. The tofu in the *L. plantarum* group and the tofu in the naturally fermented group had a more similar microstructure with a uniform and dense structure. The reason for the differences in the microstructure of the three types of tofu was probably that the tofu in the *L. plantarum* group and the tofu in the naturally fermented group could better promote soy protein gelatin and form a denser cross-linked structure, which also well-explained the differences in hardness and rheological properties of tofu. Thus, coagulants fermented by *L. plantarum* could be used as a new source of lactic acid bacteria tofu.

#### 3.3.2. Comparison of the Rheological Properties of Acidic Whey Tofu

**Figure 6:**
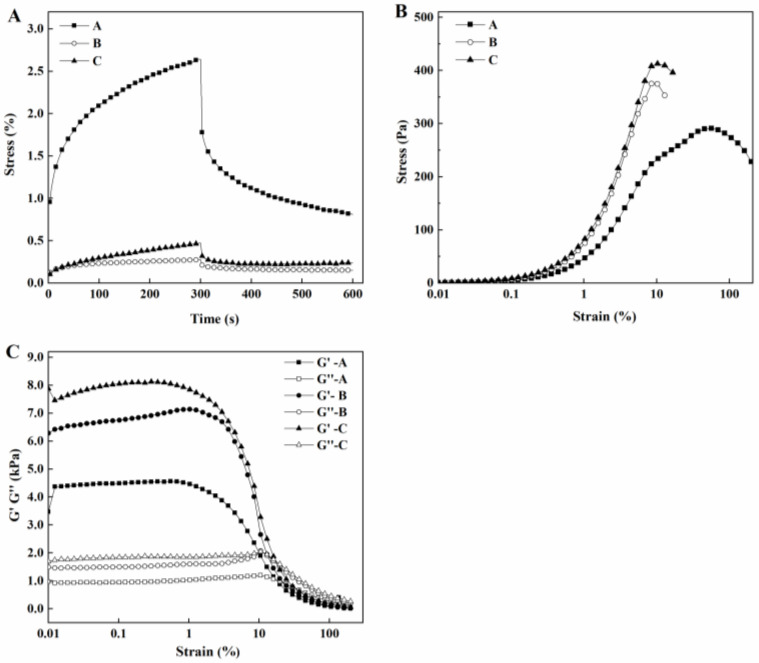
Creep-recovery curve (**A**), strain creep curves (**B**), and changes in G’ and G’’ as a function of strain (**C**) of tofu manufactured from coagulant fermented by *L. paracasei* (**A**), *L. plantarum* (**B**), and natural bacteria (**C**).

As shown in Figure 6, the shear stress of 8 Pa was applied to tofu, and a transient strain was generated in the initial stage due to the purely elastic nature of the sample. Then, the strain increased rapidly due to the viscous nature of the sample, causing relaxation elasticity. Subsequently, the strain was reduced. When the stress was withdrawn, some of the deformations could be recovered. However, still, some deformation remained, indicating the viscoelastic nature of the sample [6].

The fits for all 3 samples had R^2^ greater than 0.99, indicating good fits. As shown in Table 3, tofu in the *L. plantarum* group and the naturally fermented group had a larger G_0_ than tofu in the *L. paracasei* group, indicating that the former two had greater stiffness, which was consistent with the results of the texture measurements. The tofu in the *L. plantarum* group had the largest G_1_ and μ_0_, while the tofu in the *L. paracasei* group had the smallest G_1_ and μ_0_, which might be related to the internal forces during gelatinization.

As shown in Figure 6, the strain scans for *L. paracasei* tofu, *L. plantarum* tofu, and naturally fermented tofu showed that the shear stress increased slowly in the beginning, then increased sharply, and finally decreased rapidly as the shear strain increased. The storage and loss moduli of the three species remained essentially unchanged at low strains, indicating that the three tofu species were not damaged in this strain range and could maintain their structural stability. The tofu in the *L. plantarum* and naturally fermented groups had greater yield stress than the tofu in the *L. paracasei* group, indicating that the former two had greater gelation strength. The tofu in the *L. paracasei* group fractured at 56% of strain, while the tofu in the *L. plantarum* and naturally fermented groups fractured at about 10% of strain, indicating that the tofu from the *L. paracasei* group was more deformable. The order of fracture stress for the three types of tofu was naturally fermented group tofu > *L. plantarum* group tofu > *L. paracasei* group tofu. The order of yield strain was *L. paracasei* group tofu > *L. plantarum* group tofu > naturally fermented group tofu. Thus, although the gelation strength was higher in the tofu in the *L. plantarum* and naturally fermented groups, the gelatin became more brittle as a result.

#### 3.3.3. Comparison of the Organic Acid Content in the Acidic Whey Tofu

The contents of organic acids in the three kinds of tofu were measured. The results are shown in Table 4. Seven organic acids were detected in the three acidic tofu, namely formic acid, malic acid, lactic acid, acetic acid, citric acid, succinic acid, and propionic acid, among which contents of lactic acid, acetic acid, and propionic acid were relatively high. The total amount of organic acids in *L. paracasei* tofu, *L. plantarum* tofu, and naturally fermented tofu was 12.87 g/L, 15.81 g/L, and 15.39 g/L, respectively. The total amount of organic acids in *L. plantarum* tofu and naturally fermented tofu was similar and higher than that in *L. paracasei* tofu.

## 4. Conclusions

In this present study, acidic whey tofu was prepared using coagulants fermented by either *L. paracasei* or *L. plantarum* as the coagulant. Based on the pH, water-holding capacity, hardness, and microstructure of tofu gelatin, good-quality tofu gelatin was obtained when the ratio of coagulant was 10%, and the holding temperature was 37 °C. The quality of tofu produced by pure bacterial fermentation was compared with that produced by natural fermentation under optimal gelatin preparation conditions. It was found that the tofu produced by *L. paracasei*-fermented coagulant had higher pH, less hardness, and a rougher network structure, while the tofu produced by *L. plantarum*-fermented coagulant was closer to the tofu produced by natural fermentation in terms of pH, texture, rheology, and microstructure.

## Figures and Tables

**Table 1:** Effect of different temperatures on properties of tofu gelatin induced by coagulants fermented using *L. paracasei* and *L. plantarum*.

Temperature/°C	pH		Water-Holding Capacity (%)		Hardness (g)	
	*L. paracasei*	*L. plantarum*	*L. paracasei*	*L. plantarum*	*L. paracasei*	*L. plantarum*
27	6.34 ± 0.03 ^c^	5.51 ± 0.04 ^c^	-	76.6 ± 1.7 ^bc^	-	23 ± 1 ^b^
37	5.64 ± 0.02 ^a^	4.50 ± 0.02 ^a^	79 ± 4	70.2 ± 0.4 ^a^	20.2 ± 0.6	57 ± 2 ^c^
47	5.70 ± 0.03 ^b^	5.26 ± 0.01 ^b^	84 ± 2	75.4 ± 2.8 ^ab^	14.7 ± 2.2	41 ± 4 ^d^
57	6.39 ± 0.01 ^d^	6.18 ± 0.02 ^d^	-	80.2 ± 2.8 ^bc^	-	17 ± 2 ^a^
67	6.28 ± 0.02 ^c^	6.27 ± 0.02 ^d^	-	81.6 ± 1.6 ^c^	-	13 ± 4 ^a^

Note: ‘-’ indicates unmeasured; means followed by different letters as superscripts in a column are significantly different (*p* < 0.05).

**Table 2:** Effect of different percentages of *L. paracasei*—and *L. plantarum*—fermented coagulant on the properties of tofu gelatin.

Percentage (%)	pH		Water-Holding Capacity (%)		Hardness (g)	
	*L. paracasei*	*L. plantarum*	*L. paracasei*	*L. paracasei*	*L. plantarum*	*L. paracasei*
4	6.36 ± 0.01 ^d^	5.55 ± 0.01 ^d^	-	75.5 ± 0.3 ^a^	-	46 ± 2 ^a^
7	6.09 ± 0.02 ^c^	5.17 ± 0.02 ^c^	-	72.0 ± 1.4 ^a^	-	49 ± 3 ^ab^
10	5.64 ± 0.03 ^b^	4.50 ± 0.02 ^b^	79 ± 3 ^a^	70.2 ± 0.4 ^a^	20.2 ± 0.6 ^a^	57 ± 2 ^ab^
13	5.59 ± 0.03 ^b^	4.42 ± 0.01 ^a^	77 ± 1 ^a^	72.2 ± 2.8 ^a^	21.5 ± 0.5 ^a^	48 ± 5 ^ab^
16	5.51 ± 0.01 ^a^	4.35 ± 0.03 ^a^	76 ± 2 ^a^	70.5 ± 7.1 ^a^	21.5 ± 0.2 ^a^	54 ± 5 ^b^

Note: ‘-’ indicates unmeasured; means followed by different letters as superscripts in a column are significantly different (*p* < 0.05).

**Table 3:** pH, water-holding capacity, TPA parameters, and parameters of Burger’s model for the creep behavior of tofu manufactured from coagulants fermented by *L. paracasei*, *L. plantarum*, and natural bacteria.

Index	*L. paracasei*	*L. plantarum*	Natural Bacteria
pH	5.62 ± 0.03 ^c^	4.64 ± 0.04 ^b^	4.23 ± 0.03 ^a^
Water-holding capacity (%)	89.8 ± 1.4 ^a^	92.1 ± 0.8 ^b^	88.6 ± 0.8 ^a^
Hardness (g)	212 ± 5 ^a^	338 ± 38 ^b^	337 ± 21 ^b^
Springiness	0.93 ± 0.02 ^a^	0.92 ± 0.01 ^a^	0.93 ± 0.03 ^a^
Cohesiveness	0.83 ± 0.01 ^b^	0.79 ± 0.01 ^a^	0.79 ± 0.01 ^a^
Chewingness (g)	164 ± 1 ^a^	246 ± 26 ^b^	247 ± 9 ^b^
G_0_/×10^3^ Pa	0.81 ± 0.01 ^a^	6.49 ± 0.07 ^b^	7.76 ± 0.13 ^c^
G_1_/×10^3^ Pa	0.85 ± 0.01 ^a^	9.12 ± 0.14 ^c^	6.93 ± 0.11 ^b^
λ/s	36.8 ± 1.3 ^b^	27.7 ± 0.9 ^a^	38.3 ± 1.5 ^b^
μ_0_/×10^5^ Pa	3.23 ± 0.08 ^a^	36.34 ± 0.66 ^c^	9.43 ± 0.09 ^b^

Note: Means followed by different letters as superscripts in the same arrow are significantly different (*p* < 0.05). G_0_ indicated the hardness of tofu. G_1_ indicated the cohesion of tofu. λ indicated the time required to reach the maximum deformation. μ_0_ indicated the viscosity of tofu.

**Table 4:** Organic acids in tofu manufactured from coagulant fermented by *L. paracasei* and *L. plantarum* and natural bacteria.

Organic Acids/g/L	*L. paracasei*	*L. plantarum*	Natural Bacteria
Formic acid	0.07 ± 0.01 ^a^	0.12 ± 0.01 ^b^	0.15 ± 0.01 ^c^
Malic acid	0.24 ± 0.01 ^c^	0.18 ± 0.00 ^b^	0.11 ± 0.02 ^a^
Lactic acid	3.60 ± 0.09 ^a^	6.37 ± 0.01 ^b^	3.72 ± 0.03 ^a^
Acetic acid	1.70 ± 0.02 ^a^	1.98 ± 0.02 ^b^	2.43 ± 0.02 ^c^
Citric acid	0.19 ± 0.01 ^a^	0.24 ± 0.03 ^b^	0.19 ± 0.01 ^a^
Succinic acid	0.21 ± 0.00 ^a^	0.23 ± 0.01 ^b^	0.73 ± 0.00 ^c^
Propionic acid	6.86 ± 0.01 ^a^	6.70 ± 0.01 ^a^	8.06 ± 0.77 ^a^
Total contents	12.87 ± 0.01 ^a^	15.81 ± 0.01 ^b^	15.39 ± 0.75 ^b^

Note: Means followed by different letters as superscripts in the same arrow are significantly different (*p* < 0.05).

## Data Availability

Data is contained within the article or Supplementary Materials.

## Supplementary Materials

The following supporting information can be downloaded at: https://www.mdpi.com/article/10.3390/foods12050918/s1, Figure S1: The processing procedure for the manufacture of acidic whey tofu; Figure S2: Effect of different temperatures on tofu gelatin induced by coagulant fermented using *L. paracasei* (A) and *L. plantarum* (B); Figure S3: Effect of amount of coagulant fermented using *L. paracasei* (A) and *L. plantarum* (B) on tofu gelatin; Table S1: Parameters of rheological properties for tofu gelatin induced by coagulant fermented using *L. paracasei* and *L. plantarum*.

## References

[1] Lakshmanan R., De Lamballerie M., Jung S. (2006). Effect of soybean-to-water ratio and pH on pressurized soymilk properties. J. Food Sci..

[2] Yagasaki K., Kousaka F., Kitamura K. (2000). Potential improvement of soymilk gelation properties by using soybeans with modified protein subunit compositions. Breed. Sci..

[3] Wei G., Wang K., Liu Y., Regenstein J.M., Liu X., Zhou P. (2019). Characteristic of low-salt solid-state fermentation of Yunnan oil furu with *Mucor racemosus*: Microbiological, biochemical, structural, textural and sensory properties. Int. J. Food Sci. Tech..

[4] Chua J.-Y., Liu S.-Q. (2019). Soy whey: More than just wastewater from tofu and soy protein isolate industry. Trends Food Sci. Tech..

[5] Zhang Q., Li W., Feng M., Dong M. (2013). Effects of different coagulants on coagulation behavior of acid-induced soymilk. Food Hydrocoll..

[6] Li C., Rui X., Zhang Y., Cai F., Chen X., Jiang M. (2017). Production of tofu by lactic acid bacteria isolated from naturally fermented soy whey and evaluation of its quality. LWT-Food Sci. Technol..

[7] Ringgenberg E., Alexander M., Corredig M. (2013). Effect of concentration and incubation temperature on the acid induced aggregation of soymilk. Food Hydrocoll..

[8] Ali F., Tian K., Wang Z.-X. (2021). Modern techniques efficacy on tofu processing: A review. Trends Food Sci. Tech..

[9] Rekha C.R., Vijayalakshmi G. (2013). Influence of processing parameters on the quality of soycurd (tofu). J. Food Sci. Technol..

[10] Chen C., Rui X., Lu Z., Li W., Dong M. (2014). Enhanced shelf-life of tofu by using bacteriocinogenic *Weissella hellenica* D1501 as bioprotective cultures. Food Control.

[11] Riciputi Y., Serrazanetti D.I., Verardo V., Vannini L., Caboni M.F., Lanciotti R. (2016). Effect of fermentation on the content of bioactive compounds in tofu-type products. J. Funct. Foods.

[12] Serrazanetti D.I., Ndagijimana M., Miserocchi C., Perillo L., Guerzoni M.E. (2013). Fermented tofu: Enhancement of keeping quality and sensorial properties. Food Control.

[13] Wang Y.-J., Chen X.-H., Li W., Jiang M., Rui X., Dong M.-S. (2014). Textural Properties and Microstructure of Tofu Coagulated by Plum Juice. Food Sci..

[14] Yuan S., Chang S.K. (2007). Texture profile of tofu as affected by instron parameters and sample preparation, and correlations of instron hardness and springiness with sensory scores. J. Food Sci..

[15] Zhao H., Wang Y., Li W., Qin F., Chen J. (2017). Effects of oligosaccharides and soy soluble polysaccharide on the rheological and textural properties of calcium sulfate-induced soy protein gels. Food Bioprocess Tech..

[16] Bi C.-H., Li D., Wang L.-J., Gao F., Adhikari B. (2014). Effect of high shear homogenization on rheology, microstructure and fractal dimension of acid-induced SPI gels. J. Food Eng..

[17] Kohyama K., Sano Y., Doi E. (1995). Rheological characteristics and gelation mechanism of tofu (soybean curd). J. Agric. Food Chem..

[18] Noh E., Park S., Pak J., Hong S., Yun S. (2005). Coagulation of soymilk and quality of tofu as affected by freeze treatment of soybeans. Food Chem..

[19] Zhu Q., Wu F., Saito M., Tatsumi E., Yin L. (2016). Effect of magnesium salt concentration in water-in-oil emulsions on the physical properties and microstructure of tofu. Food Chem..

[20] Kao F.J., Su N.W., Lee M.H. (2003). Effect of calcium sulfate concentration in soymilk on the microstructure of firm tofu and the protein constitutions in tofu whey. Agric. Food Chem..

[21] Wang R., Guo S. (2016). Effects of endogenous small molecular compounds on the rheological properties, texture and microstructure of soymilk coagulum: Removal of phytate using ultrafiltration. Food Chem..

[22] Lin H.F., Lu C.P., Hsich J.F., Kuo M.I. (2016). Effect of ultrasonic treatment on the rheological property and microstructure of tofu made from different soybean cultivars. Innov. Food Sci. Emerg. Technol..

[23] Dickinson E. (2012). Emulsion gels: The structuring of soft solids with protein-stabilized oil droplets. Food Hydrocoll..

[24] Veronicaa O. (2008). Effect of different coagulants on yield and quality of tofu from soymilk. Eur. Food Res. Technol..

[25] Zuo F., Chen Z., Shi X., Wang R., Guo S. (2016). Yield and textural properties of tofu as affected by soymilk coagulation prepared by a high-temperature pressure cooking process. Food Chem..

[26] Qin X.-S., Luo S.-Z., Cai J., Zhong X.-Y., Jiang S.-T., Zhao Y.-Y., Zheng Z. (2016). Transglutaminase-induced gelation properties of soy protein isolate and wheat gluten mixtures with high intensity ultrasonic pretreatment. Ultrason. Sonochem..

[27] Sun X.D., Arntfield S.D. (2012). Molecular forces involved in heat-induced pea protein gelation: Effects of various reagents on the rheological properties of salt-extracted pea protein gels. Food Hydrocoll..

[28] Naji-Tabasi S., Razavi S.M.A. (2017). New studies on basil (*Ocimum bacilicum* L.) seed gum: Part III–Steady and dynamic shear rheology. Food Hydrocoll..

