# Supplementary material for: Effect of Coagulant and Treatment Conditions on the Gelation and Textural Properties of Acidic Whey Tofu
Source: Foods. 2023 Feb 21;12(5):918. doi: 10.3390/foods12050918 (PMC10000490; doi:10.3390/foods12050918)
Supplement: Supplementary file 1 [file foods-12-00918-s001.zip › foods-2178996-supplementary.pdf]

Supplementary

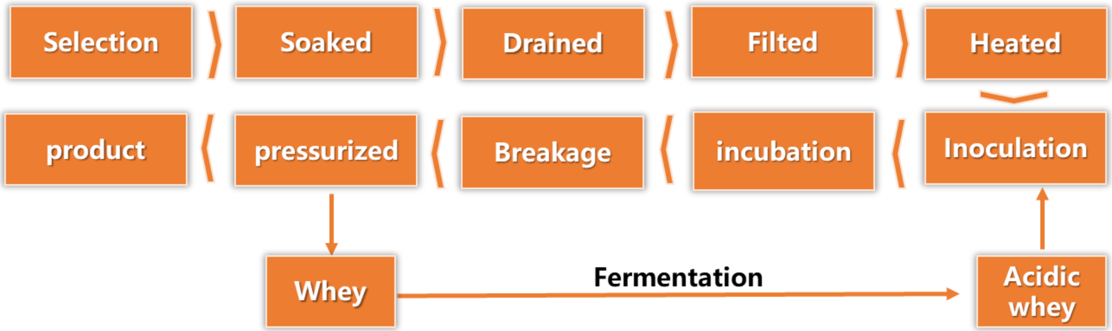

Figure S1. The processing procedure for the manufacture of acidic whey tofu.

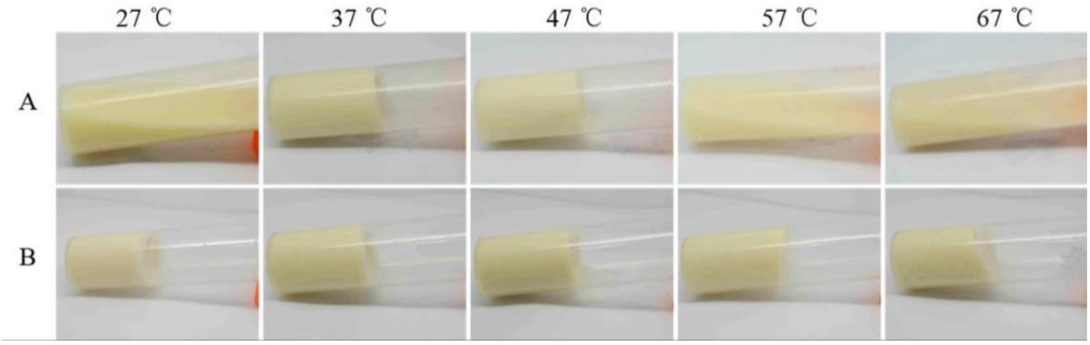

Figure S2. Effect of different temperatures on tofu gelatin induced by coagulant fermented using *L. paracasei* (**A**) and *L. plantarum* (**B**).

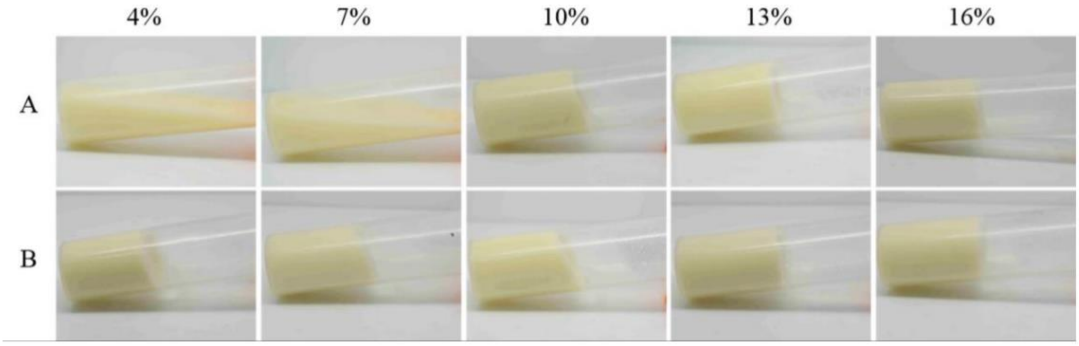

Figure S3. Effect of amount of coagulant fermented using *L. paracasei* (**A**) and *L. plantarum* (**B**) on tofu gelatin.

Table S1. Parameters of rheological properties for tofu gelatin induced by coagulant fermented using *L. paracasei* and *L. plantarum*.

Parameter	*L. paracasei*	*L. plantarum*
Gel time/min	182 ± 4	133 ± 2
Storage modulus G'_{max}/Pa	470 ± 20	1250 ± 50
$G' \propto \omega^n$ n	0.1156 ± 0.0003	0.1220 ± 0.0006
$G' \propto \omega^n$ R^2	0.9985 ± 0.0004	0.9993 ± 0.0003
